# Decreased Platelet Aggregation in Patients with Decompensated Liver Cirrhosis and TIPS Implantation

**DOI:** 10.3390/biomedicines11072057

**Published:** 2023-07-21

**Authors:** Asala Nassar, Jan Patrick Huber, Daniela Stallmann, Diana Sharipova, Muataz Ali Hamad, Michael Schultheiss, Robert Thimme, Daniel Duerschmied, Rüdiger Eberhard Scharf, Dominik Bettinger, Krystin Krauel

**Affiliations:** 1 Department of Medicine II, Medical Center University of Freiburg, Faculty of Medicine, University of Freiburg, D-79106 Freiburg, Germany; 2 Department of Cardiology and Angiology I, Heart Center, University of Freiburg, D-79106 Freiburg, Germany; 3 Spemann Graduate School of Biology and Medicine (SGBM), University of Freiburg, D-79104 Freiburg, Germany; 4 Faculty of Biology, University of Freiburg, D-79104 Freiburg, Germany; 5 Berta-Ottenstein-Program, Faculty of Medicine, University of Freiburg, D-79106 Freiburg, Germany; 6 Department of Cardiology, Angiology, Haemostaseology and Medical Intensive Care, University Medical Center Mannheim, Medical Faculty Mannheim, Heidelberg University, D-68167 Mannheim, Germany; 7 European Center for AngioScience (ECAS) and German Center for Cardiovascular Research (DZHK), Partner Site Heidelberg/Mannheim, D-68167 Mannheim, Germany; 8 Program in Cellular and Molecular Medicine, Boston Children’s Hospital, Harvard Medical School, Boston, MA 02115, USA; 9 Division of Experimental and Clinical Hemostasis, Hemotherapy, and Transfusion Medicine, Blood and Hemophilia Comprehensive Care Center, Institute of Transplantation Diagnostics and Cell Therapy, Heinrich Heine University Medical Center, D-40225 Düsseldorf, Germany

**Keywords:** liver cirrhosis, TIPS implantation, platelets, platelet aggregation

## Abstract

Transjugular intrahepatic portosystemic shunt (TIPS) implantation is an effective treatment of portal hypertension in patients with decompensated liver cirrhosis. However, some patients develop TIPS thrombosis with recurrence of portal hypertension. The role of platelets in TIPS thrombosis and the necessity of antiplatelet therapy is unclear. Therefore, we aimed to study platelet function in patients with liver cirrhosis prior to and after TIPS implantation. Platelet aggregation was tested in peripheral and portal-vein blood patient samples on the day (D) of TIPS implantation (D0), D4 and D30 following the procedure (platelet count above 100 × 10^3^/µL, aspirin starting on D5) using whole-blood impedance aggregometry (WBIA) and light transmission aggregometry (LTA). In addition, surface platelet activation markers (P-selectin, activated GPIIb/IIIa) and platelet–neutrophil complexes (PNCs) were assessed by flow cytometry. Thrombin receptor activating peptide 6 (TRAP-6), adenosine diphosphate (ADP) and arachidonic acid (AA) were used as agonists. Healthy subjects were included as controls. Agonist-induced platelet aggregation was reduced (WBIA: TRAP-6 *p* < 0.01, ADP *p* < 0.01, AA *p* < 0.001; LTA: TRAP-6 *p* = 0.13, ADP *p* = 0.05, AA *p* < 0.01) in patients (D0, n = 13) compared with healthy subjects (n = 9). While surface activation markers at baseline were negligibly low, the percentage of PNCs was higher in patients than in controls (*p* < 0.05). ADP-induced P-selectin expression was increased (*p* < 0.001), whereas TRAP-6-induced GPIIb/IIIa activation was impaired (*p* < 0.001) in patients versus controls. PNC formation in response to agonists was not different between groups. Results did not differ between peripheral and portal-vein blood of patients (D0, n = 11) and did not change over time (D0, D4, D30) following TIPS implantation (n = 9). In summary, patients with decompensated liver cirrhosis display in vitro platelet aggregation defects in response to various agonists. Defective aggregation persists upon TIPS implantation. Therefore, we conclude that antiplatelet treatment to prevent TIPS thrombosis is questionable.

## 1. Introduction

Liver cirrhosis is a major healthcare problem worldwide, with a high rate of morbidity and mortality [1,2]. Both morbidity and mortality are caused by complications of liver cirrhosis, which are mainly related to the development of portal hypertension [3,4]. Frequent complications of portal hypertension are variceal bleeding and development of ascites with the need for diuretic treatment and recurrent large-volume paracenteses [5]. Implantation of a transjugular intrahepatic portosystemic shunt (TIPS) has emerged as an effective and safe interventional treatment option for patients with decompensated cirrhosis [6]. During the last decade, there have been many technical improvements in TIPS implantation, especially the development of covered stents [6,7]. The use of covered stents has markedly reduced stent stenosis and thrombosis. Nowadays, TIPS dysfunction is mainly due to technical aspects (e.g., damage of the covering of the stent) or caused by hemodynamical changes within the stent (insufficient position in the liver vein [8]), leading to a non-laminar flow and, subsequently, to the development of stenosis or thrombosis. However, in comparison to patients with coronary artery disease and stent implantation, an intervention in which platelets play a crucial role in the pathogenesis of stent thrombosis, the role of platelets in TIPS dysfunction is unknown. Importantly, there are no general recommendations on antithrombotic prophylaxis following TIPS implantation, and anticoagulation or platelet inhibition strategies vary significantly between centers [9]. One reason why platelet inhibition is not routinely performed may be that about 75% of patients with liver cirrhosis are thrombocytopenic, displaying platelet counts of <150 × 10^3^/µL or less. Moreover, there are conflicting findings concerning platelet function and activity ex vivo in patients with liver cirrhosis. For example, several studies documented enhanced platelet aggregation, whereas other studies reported on reduced platelet aggregation [10,11]. Taken together, it appears to be essential to perform in-depth analyses of the functional status of platelets prior to and after TIPS implantation before special recommendations for pharmacological prophylaxis of TIPS dysfunction can be made. Therefore, the aim of this study was to characterize platelet function prior to TIPS implantation and within the first 30 days after intervention in patients with decompensated cirrhosis.

## 2. Materials and Methods

### 2.1. Study Design

The study was conducted in accordance with the Declaration of Helsinki and approved by the Ethics Committee of the University of Freiburg (EK 486/19; 19 December 2019). Informed written consent was obtained from all participants prior to enrollment. Between October 2020 and September 2021, we recruited 14 consecutive patients with decompensated liver cirrhosis who were scheduled for TIPS implantation at the University Medical Center Freiburg. Inclusion criteria were: (1) decompensated liver cirrhosis with indication for TIPS and (2) platelet count > 100 × 10^3^/µL. The severity of cirrhosis was assessed using the Child–Pugh score (Table 1) [12]. Patients were also classified according to their mortality risk after TIPS implantation according to the Freiburg index of post-TIPS survival (FIPS) [13]. Laboratory parameters were assessed in the Freiburg Central laboratory (Table 1). Hirudinized (S-Monovette Hirudin 1.6 mL; Sarstedt, Nümbrecht, Germany) and citrated (S-Monovette citrate 3.2% 10 mL; Sarstedt, Nümbrecht, Germany) peripheral blood was obtained on the day of TIPS implantation before the procedure (D0), four days (D4) and 30 days (D30) after the intervention. In addition to the peripheral blood sample on D0, blood was also collected from the portal vein during the intervention. Patients were released on D4, received aspirin (100 mg/day), starting on D5, and had a follow-up on D30, including evaluation of the clinical response to TIPS implantation. Patients with no further variceal bleeding and no further paracenteses within D30 after TIPS implantation were defined as clinical responders to TIPS implantation. Color Doppler ultrasound was performed on D30 to assess shunt function. Flow velocity of <80 cm/s in the TIPS stent and/or flow velocity < 30 cm/s in the extrahepatic portal vein were used as criteria for shunt dysfunction [14].

**Figure 1:**
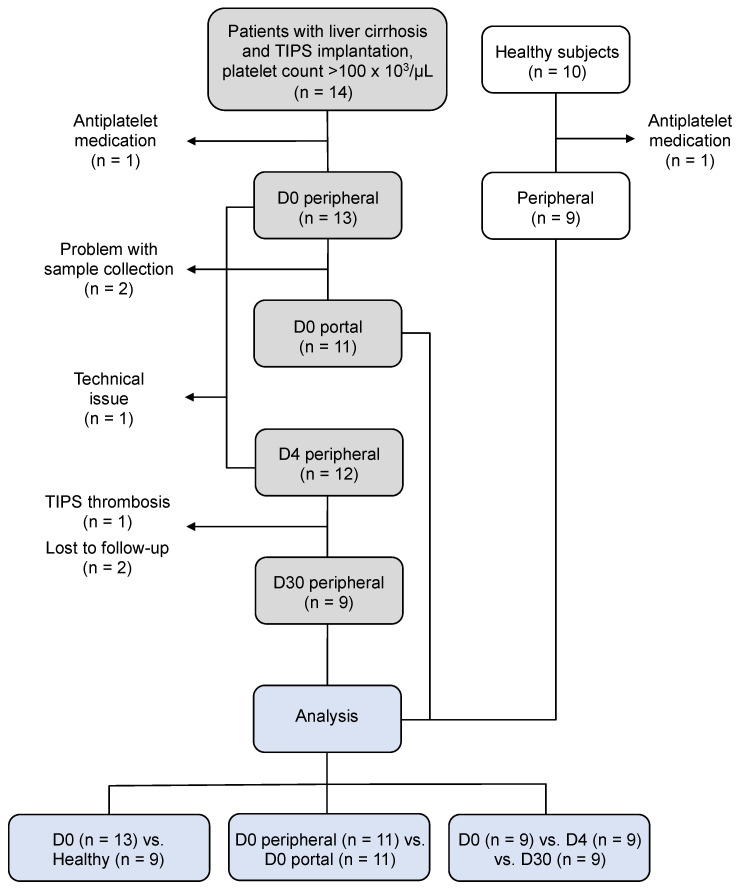
Flow chart of the study population. We recruited 14 patients with liver cirrhosis scheduled for TIPS implantation and platelet counts > 100 × 10^3^/μL. One patient was excluded because of aspirin therapy that started before the procedure. We further excluded patients due to problems with collecting portal-vein blood samples (n = 2), technical issues during the measurement (n = 1) and the development of TIPS thrombosis between D4 and D30 (n = 1). Two patients did not return for follow-up on D30. In addition, we collected 10 peripheral blood samples from healthy subjects. One volunteer had to be excluded because of aspirin medication. For the final analysis, (1) 13 patients before the TIPS implantation were compared with 9 healthy subjects, (2) paired comparison for the sample’s origin was performed with 11 patients, and (3) longitudinal paired analysis was performed with 9 patients.

Ten healthy subjects were recruited from the medical staff and served as controls. Exclusion criteria are displayed and specified in Figure 1.

### 2.2. Whole-Blood Impedance Aggregometry (WBIA)

Platelet aggregation was performed in hirudinized blood using the Multiplate Analyzer from Roche Diagnostics following the manufacturer’s instructions. Thrombin receptor activating peptide 6 (TRAP-6, 32 µM; TRAPtest, Roche Diagnostics, Rotkreuz, Switzerland), adenosine diphosphate (ADP, 6.5 µM; ADPtest, Roche Diagnostics, Rotkreuz, Switzerland), and arachidonic acid (AA, 0.5 mM; ASPItest, Roche Diagnostics, Rotkreuz, Switzerland) were used as agonists. The results are provided as area under the curve (AUC) in arbitrary units (U).

### 2.3. Light Transmission Aggregometry (LTA)

In addition, platelet aggregation was analyzed using the platelet aggregation profiler PAP-8 from möLab according to the manufacturer’s instructions. In brief, platelet-rich plasma (PRP) was generated from citrated blood by centrifugation (150× *g*, 10 min, room temperature [RT]). After removing PRP, platelet-poor plasma (PPP) was obtained by a second centrifugation step (1500× *g*, 15 min, RT). PPP was used to set 100% light transmission. Platelet aggregation was induced by adding TRAP-6 amide (34 µM; möLab, Langenfeld, Germany), ADP (20 µM; möLab, Langenfeld, Germany) or AA (1.6 mM; möLab, Langenfeld, Germany) to PRP and was tracked for 10 min. The results are provided as maximal aggregation in percent.

### 2.4. Flow Cytometry

We used flow cytometry to measure surface P-selectin, activated GPIIb/IIIa and PNCs. Citrated blood was diluted 1:6 with Dulbecco phosphate-buffered saline (DPBS with Ca^2+^/Mg^2+^) containing 525 antithrombin units/mL of recombinant hirudin (Hyphen BioMed, Neuville-sur-Oise, France) and incubated (15 min, RT) with TRAP-6 (20 µM; Abcam, Cambridge, UK), ADP (20 µM; möLab, Langenfeld, Germany) or DPBS. Next, samples were incubated (15 min, RT) with two specific antibody mixes: (1) HIP1 clone mouse anti-human CD42b-PE + AK4 clone mouse anti-human CD62P-PE Cy7 + PAC-1 clone mouse anti-human CD41/CD61-AF647; (2) HI30 clone mouse anti-human CD45-Pacific Blue + HIP1 clone mouse anti-human CD42b-PE; or matched isotype control mixes (all from BioLegend, San Diego, CA, USA). Subsequently, samples were incubated (30 min, RT) with prewarmed (37 °C) phosflow lyse/fix buffer (BD Biosciences, San Jose, CA, USA), centrifuged (700× *g*, 5 min, RT), resuspended with DPBS and analyzed using a FACSCanto II flow cytometer (BD, Franklin Lakes, NJ, USA). The results are provided as the percentage of positive cells.

### 2.5. Statistical Analysis

GraphPad Prism version 9 (GraphPad Software, San Diego, CA, USA) was used for statistical analysis. Data distribution was assessed using the Shapiro–Wilk test. For normally distributed data, we performed unpaired (D0, patients versus healthy subjects) and paired (D0, peripheral versus portal venous blood) two-tailed *t*-test or repeated measures one-way analysis of variance with Tukey multiple comparison test (D0 versus D4 versus D30). For not normally distributed data, we performed the Mann–Whitney test (D0, patients versus healthy subjects), Wilcoxon test (D0 peripheral versus portal venous blood) or Friedman test with Dunn multiple comparison test (D0 versus D4 versus D30). Data are presented as mean ± SD (normal distribution) or median with interquartile range (non-normal distribution). Statistical significance was considered with a two-tailed *p* < 0.05.

## 3. Results

### 3.1. Patient Characteristics

Baseline characteristics of the patients enrolled are shown in Table 1. Thirteen patients who were allocated to TIPS implantation due to decompensated liver cirrhosis were included in this study. Of the 13 patients, 11 (84.6%) had alcoholic cirrhosis, 1 patient had hemochromatosis and another 1 suffered from primary biliary cholangitis. The predominant indication for TIPS implantation was recurrent ascites in 8 patients (61.5%), and 5 patients received TIPS for secondary prophylaxis of variceal bleeding. Of the 13 patients, 7 were in Child–Pugh stage B. Further, 12 patients were classified as FIPS low risk, and only 1 patient received TIPS implantation in a FIPS high-risk situation. Of the 13 patients, 5 (38.5%) had platelet counts < 150 × 10^3^/µL.

During follow-up, one patient developed TIPS thrombosis, possibly due to insufficient stent position in the liver vein (Figure S1). Nine patients had complete follow-up on D30. Eight of them showed clinical response to TIPS implantation and displayed no sonographic signs of TIPS dysfunction. One patient had persisting ascites after TIPS implantation and was considered a non-responder. However, no TIPS dysfunction was detected during ultrasound assessment, indicative of no shunt-related response failure. Non-response was explained by more advanced liver disease and long-standing decompensation.

### 3.2. Platelet Aggregation Is Decreased in Patients with Liver Cirrhosis Prior to TIPS Implantation

**Figure 2:**
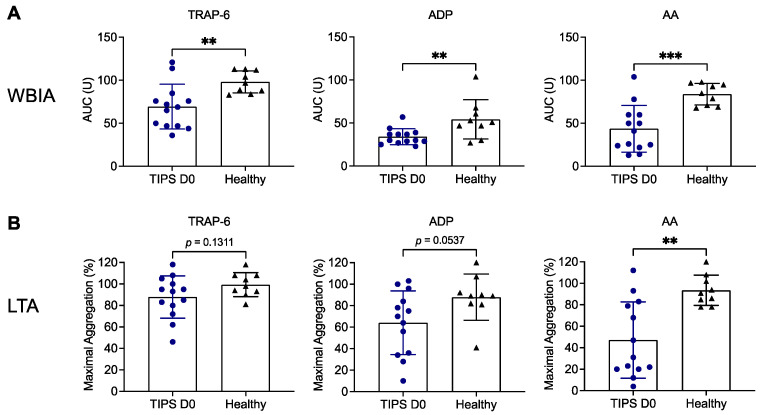
Platelet aggregation testing in patients with liver cirrhosis prior to TIPS implantation compared to healthy volunteers. Platelet aggregation was assessed in hirudinized blood (1:2 diluted) (**A**) and citrated blood-derived platelet-rich plasma (**B**) from patients with liver cirrhosis (n = 13, blue circles) and healthy subjects (black triangles, n = 9) in response to TRAP-6, ADP or AA, using WBIA and LTA, respectively. Results are presented as AUC in arbitrary units (**A**) or as percentage of maximal aggregation (**B**). ** *p* < 0.01; *** *p* < 0.001. AA, arachidonic acid; ADP, adenosine diphosphate; AUC, area under the curve; D0, Day 0; LTA, light transmission aggregometry; TIPS, transjugular intrahepatic portosystemic shunt; TRAP-6, thrombin receptor activating peptide 6; WBIA, whole-blood impedance aggregometry.

First, we assessed platelet function in patients with liver cirrhosis prior to TIPS implantation (TIPS D0) compared to healthy subjects upon stimulation with various platelet agonists. Using WBIA, we detected significantly decreased platelet aggregation in patients in response to TRAP-6 (69.4 ± 26.1 U versus 98.1 ± 12.7 U; *p* = 0.0063), ADP (34.2 ± 9.2 U versus 54.2 ± 22.8 U; *p* = 0.0093) or AA (43.6 ± 27.0 U versus 83.9 ± 12.5 U; *p* = 0.0005) (Figure 2A). Platelet aggregation in response to AA (47.2% ± 35.5% versus 93.6% ± 14.1%; *p* = 0.0014) was also significantly reduced in the LTA method, while ADP-induced (64.2% ± 29.7% versus 87.9% ± 21.5%; *p* = 0.0537) or TRAP-6-induced (87.9% ± 19.7% versus 99.3% ± 11.3%; *p* = 0.1311) platelet aggregation was only numerically diminished (Figure 2B).

**Figure 3:**
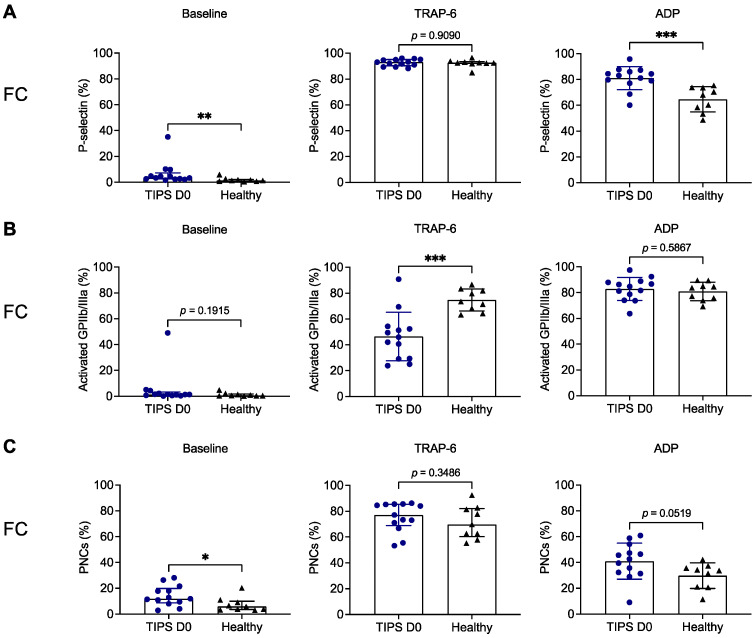
Flow cytometric analysis of platelet activation markers and platelet–neutrophil complexes in patients with liver cirrhosis prior to TIPS implantation compared to healthy volunteers. Surface expression of P-selectin (**A**), activated GPIIb/IIIa (**B**) and PNCs (**C**) was determined in citrated blood (1:6 diluted) from patients with liver cirrhosis (n = 13, blue circles) and healthy subjects (black triangles, n = 9) at baseline or in response to TRAP-6 or ADP using flow cytometry. Results are presented as percentage of positive cells. * *p* < 0.05; ** *p* < 0.01; *** *p* < 0.001. ADP, adenosine diphosphate; FC, flow cytometry; D0, Day 0; PNCs, platelet–neutrophil complexes; TIPS, transjugular intrahepatic portosystemic shunt; TRAP-6, thrombin receptor activating peptide 6.

In parallel, we assessed surface P-selectin expression and GPIIb/IIIa activation using flow cytometry. Although platelets from patients showed significantly higher surface P-selectin expression without stimulation in vitro (baseline) compared with healthy volunteers (3.4% [2.5–7.3%] versus 1.3% [0.8–2.2%]; *p* = 0.0026), the percentage of pre-activated platelets was negligibly low (Figure 3A). In addition, GPIIb/IIIa activation was below 1.5% (median) at baseline in patients and healthy subjects and not significantly different (*p* = 0.1915) (Figure 3B). The percentage of PNCs was slightly increased in patients versus controls without stimulation in vitro (11.9% [8.8–19.9%] versus 5.9% [3.6–10.0%]; *p* = 0.0304) (Figure 3C). Surprisingly, surface P-selectin expression in platelets from patients was significantly enhanced in response to ADP (81.1% ± 9.0% versus 64.7% ± 9.8%; *p* = 0.0006) but not upon stimulation with TRAP-6 (median > 90% in both groups; *p* = 0.9090) (Figure 3A). By contrast, GPIIb/IIIa activation was impaired in platelets from patients after incubation with TRAP-6 (46.4% ± 18.8% versus 74.7% ± 8.5%; *p* = 0.0004), while not affected in response to ADP (mean > 80% in both groups; *p* = 0.5867) (Figure 3B). Similarly to P-selectin surface expression upon stimulation in vitro, PNC formation was not different in response to TRAP-6 (median ≥ 70% in both groups; *p* = 0.3486) but numerically increased in patient samples that were activated with ADP (41.0% ± 14.0% versus 29.8% ± 9.9%; *p* = 0.0519) (Figure 3C).

### 3.3. Results Do Not Differ between Peripheral and Portal-Vein Blood of Patients with Liver Cirrhosis Prior to TIPS Implantation

**Figure 4:**
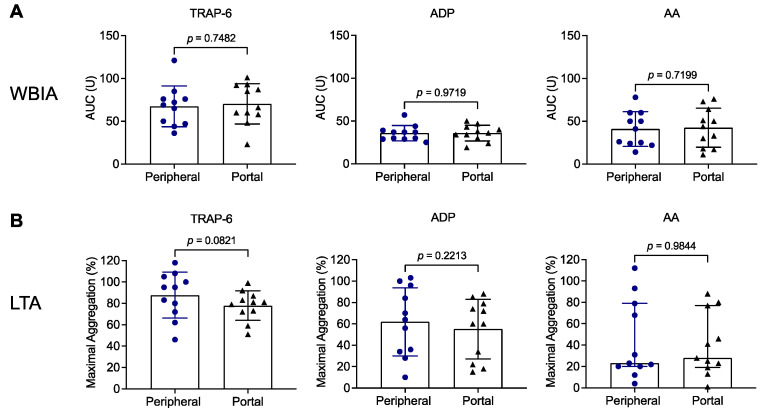
Platelet aggregation testing in patients with liver cirrhosis prior to TIPS implantation, using peripheral blood in comparison to portal-vein blood. Platelet aggregation was assessed in hirudinized blood (1:2 diluted) (**A**) and citrated blood-derived platelet-rich plasma (**B**) from patients with liver cirrhosis (n = 11, blue circles: peripheral blood, black triangles: portal-vein blood) in response to TRAP-6, ADP or AA, using WBIA and LTA, respectively. Results are presented as AUC in arbitrary units (**A**) or as percentage of maximal aggregation (**B**). AA, arachidonic acid; ADP, adenosine diphosphate; AUC, area under the curve; LTA, light transmission aggregometry; TRAP-6, thrombin receptor activating peptide 6; WBIA, whole-blood impedance aggregometry.

We next tested whether results obtained with patient samples derived from peripheral blood prior to TIPS implantation (TIPS D0) were different or similar in comparison to patient samples obtained from the portal vein during the procedure. In fact, we did not detect any significant differences between both types of samples in aggregation studies using WBIA (Figure 4A) or LTA (Figure 4B). Likewise, no differences between peripheral and portal-vein blood samples were found in flow cytometric studies (Figure S2).

### 3.4. Platelet Aggregation Remains Impaired throughout the 30-Day Follow-Up after TIPS Implantation

**Figure 5:**
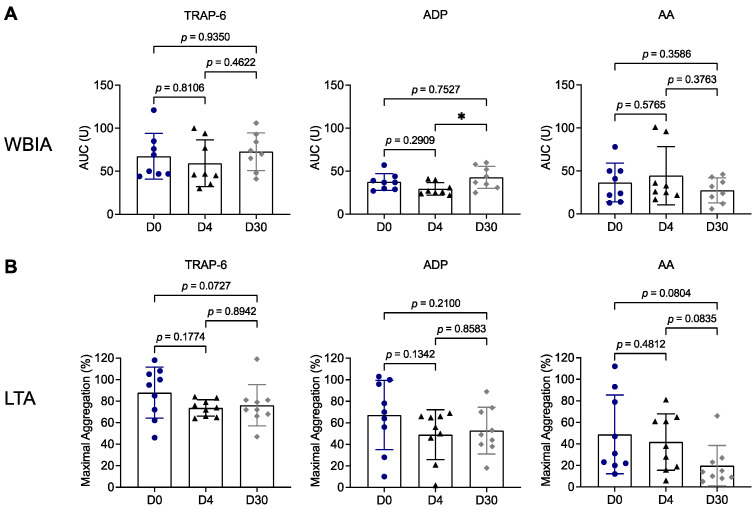
Longitudinal analysis of platelet aggregation in patients with liver cirrhosis and TIPS implantation. Platelet aggregation was assessed in hirudinized blood (1:2 diluted) (**A**) and citrated blood-derived platelet-rich plasma (**B**) from patients with liver cirrhosis (n = 9) prior to (blue circles: Day 0) and after TIPS implantation (black triangles: Day 4, gray diamonds: Day 30) in response to TRAP-6, ADP or AA, using WBIA and LTA, respectively. Results are presented as AUC in arbitrary units (**A**) or as percentage of maximal aggregation (**B**). * *p* < 0.05. AA, arachidonic acid; ADP, adenosine diphosphate; AUC, area under the curve; D0, Day 0; D4, Day 4; D30, Day 30; LTA, light transmission aggregometry; TRAP-6, thrombin receptor activating peptide 6; WBIA, whole-blood impedance aggregometry.

For longitudinal analysis, we compared platelet function in patient samples that were available for all three time points: prior to TIPS implantation (D0), D4 and D30 following the procedure. Overall, platelet aggregation examined by WBIA (Figure 5A) or LTA (Figure 5B) was not different at these three time points. We detected numerically reduced values in the LTA method with AA at D30 (19.7% ± 18.8%) versus D0 (48.8% ± 36.7%; *p* = 0.0804) and versus D4 (41.7% ± 26.2%; *p* = 0.0835). These findings can be explained by aspirin treatment of patients from D5 on.

Notably, flow cytometry studies revealed slightly but significantly reduced surface P-selectin expression in patient samples after TIPS implantation upon stimulation in vitro with TRAP-6 (D0: 92.9% ± 2.5% versus D4: 87.6% ± 5.1%; *p* = 0.0313 and versus D30: 88.3% ± 3.5%; *p* = 0.0010) or with ADP (D0: 79.4% ± 10.3% versus D30: 70.7% ± 12.1%; *p* = 0.0426) (Figure S3). However, GPIIb/IIIa activation and PNCs were not significantly different within the observation period (Figure S3).

## 4. Discussion

The role of platelets in developing TIPS thrombosis is unclear. Nonetheless, in several centers, antiplatelet medication is routinely administered after TIPS implantation, although there is no clinical or experimental evidence of such a therapy [9]. Therefore, we set out to investigate the function of platelets in patients with decompensated liver cirrhosis shortly before TIPS implantation, as well as four days and one month following the intervention. The key findings of our study indicate that platelet aggregation capacity is reduced in decompensated cirrhotic patients compared to healthy volunteers before TIPS implantation. These findings are identical in peripheral and portal-vein blood. Moreover, platelet aggregation remains impaired following TIPS implantation within an observation period of one month.

Platelet function and activity in patients with liver cirrhosis have been extensively studied in the past; however, the findings remain controversial, as recently reviewed [15,16,17]. Most investigators suggest a reduced platelet function in patients with chronic liver disease, and fewer reports assume platelet hyperreactivity. Platelet aggregation studies were predominantly performed using LTA while, more recently, one group studied platelet aggregation with WBIA [18,19]. We investigated platelet aggregation using both aggregometry methods in parallel. In line with the majority of previous studies, we observed impaired platelet aggregation in patients with liver cirrhosis (Figure 2). Using WBIA, platelet aggregation was significantly reduced compared to healthy subjects in response to all agonists tested. This was different with LTA, in which platelet aggregation was significantly reduced only in response to AA. However, we also observed a tendency toward lower aggregation responses with TRAP-6 and ADP using LTA. LTA is the gold standard for measuring platelet aggregation using platelet-rich plasma but is labor-intensive and requires expertise [20]. WBIA is easier, faster and enables platelet aggregation analysis under more physiological conditions using whole blood. Of note, WBIA is more affected by the platelet count than LTA [21,22,23]. These technical aspects may be an explanation for the discrepancy in results obtained with TRAP-6 and ADP. To overcome the platelet count issue, Zanetto et al. used WBIA to study platelet aggregation in patients with decompensated cirrhosis and normalized platelet aggregation results to the platelet count by calculating a “platelet aggregation to platelet count ratio” (PLT ratio) [19]. Using the PLT ratio, the authors finally concluded that platelet aggregation responses are higher in cirrhotic patients than in controls, which is in contrast to many previous studies. However, it is under debate whether this PLT ratio is useful and appropriate for interpreting platelet aggregation results using WBIA [24].

In response to AA, platelet aggregation in our study was most prominently reduced in patients with decompensated cirrhosis with a significant difference compared to healthy controls both in WBIA and LTA. This is consistent with a study describing reduced platelet aggregation responses due to impaired thromboxane A_2_ synthesis in liver cirrhosis [25].

Next, we explored the capability of platelets from cirrhotic patients to release alpha granules and to convert GPIIb/IIIa from the low affinity into the high affinity state, both influencing aggregatory responses. P-selectin expression in response to in vitro stimulation in the peripheral blood of patients was either unaffected (TRAP-6-induced) or even increased (ADP-induced) compared to healthy controls (Figure 3A). Thus, it is unlikely that the observed impaired platelet aggregation response in our patient cohort was due to an alpha granule storage pool defect, which has been described for patients with liver cirrhosis [26]. While P-selectin expression was not impaired, we found reduced TRAP-6-induced GPIIb/IIIa activation, which might explain the reduced aggregation response to TRAP-6 (Figure 3B). In line with this, impaired GPIIb/IIIa activation and reduced platelet aggregation have been shown in patients with alcoholic liver cirrhosis in response to stimulation in vitro [27]. Dysfunctional GPIIb/IIIa responses have been recently described for COVID-19 and sepsis [28,29,30]. However, except for one diagnosed dual hepatitis B/C virus infection, the patients included in this study had no other suspected or proven infection. Nevertheless, it cannot be excluded that local translocation of bacteria or bacterial products in the liver affected the reactivity of platelets [31,32].

It is conceivable that, among cirrhotic patients, reduced platelet reactivity ex vivo can result from pre-activation of platelets in vivo, which might be due to bacterial translocation or oxidative stress [31,32]. We detected only minor pre-activation signs of platelets from the peripheral blood of cirrhotic patients. This is documented by significantly increased P-selectin surface expression on platelets of patients without stimulation compared to healthy subjects (Figure 3A). However, the proportion of P-selectin positive platelets was low and insignificant for GPIIb/IIIa activation (Figure 3A,B). This finding is in line with previous studies [33,34]. PNC formation is another marker for pre-activation of platelets [35]. Although platelet surface activation markers at baseline were low, we detected increased numbers of PNCs in patients with liver cirrhosis compared to healthy subjects (Figure 3C). This observation is supported by former studies [36,37].

Then, we examined whether platelets can have been pre-activated locally due to a potentially inflammatory milieu in the portal vein that might be less detectable in the peripheral blood [31]. In this regard, Queck et al. found increased levels of soluble (s) platelet activation markers sP-selectin and sGPVI in the portal vein compared to the hepatic vein of patients with decompensated cirrhosis and TIPS [31].

In fact, our study does not reveal any differences in the pre-activation state of platelets or the number of PNCs between peripheral blood and portal-vein blood at baseline. Moreover, results were not dependent on the origin of the blood samples when platelet reactivity was tested following in vitro stimulation. This is consistent with a recent study by Brusilovskaya et al. demonstrating that platelet surface P-selectin expression, platelet surface GPIIb/IIIa activation and platelet–leukocyte complex formation, both at baseline and after stimulation in vitro, were indistinguishable in peripheral and central venous blood samples [38]. Moreover, only one other group assessed platelet aggregation using WBIA in portal-vein blood compared to peripheral blood from TIPS patients [18]. In agreement with our study (Figure 4), platelet aggregation in response to TRAP, ADP and AA was not significantly different between peripheral and portal-vein blood samples. By contrast, when the investigators accounted for the platelet count and calculated a PLT ratio, as outlined above, aggregation was considered to be increased in portal-vein blood compared to peripheral blood. However, as already discussed, calculating a PLT ratio is not an accepted method for analyzing WBIA data. Therefore, such an approach may be questioned and appears to be inappropriate unless validated and established [24].

Most patients enrolled in this study developed liver cirrhosis due to chronic alcohol abuse (Table 1). Interestingly, a direct inhibitory effect of alcohol on platelet responses has been proposed providing a potential explanation for the impaired platelet aggregation in our patient cohort [39,40,41].

Finally, we assessed the platelet activation status and platelet reactivity in the cirrhotic patients within an observation period of one month after TIPS placement. To our knowledge, this is the first study investigating platelet function prior to and after TIPS intervention. Platelet aggregation capability (Figure 5) and most platelet activation markers remained unchanged on Day 4 and Day 30 following TIPS compared to Day 0 after prophylactic administration of aspirin had been initiated on Day 5. Only P-selectin surface expression in response to stimulation in vitro with TRAP-6 or ADP decreased significantly on Day 30 following TIPS (Figure S3). This could be due to the successful treatment of portal hypertension verified on Day 30. Currently, we cannot explain why reduced platelet aggregation responses did not return to normal following TIPS implantation. While aspirin treatment likely accounted for the further decrease in platelet aggregation upon AA stimulation on Day 30, it should not have had a major effect on platelet aggregation in response to TRAP-6 or ADP. One can only speculate that either the time period of 30 days was not long enough to detect changes after TIPS intervention or portal hypertension was not the underlying cause of impaired platelet aggregation. This needs to be clarified in future studies.

As platelet aggregation remained reduced on Day 4 and Day 30, it might be worth revisiting whether antiplatelet medication benefits cirrhotic patients after TIPS insertion. As a standard medication in our hospital, patients received 100 mg aspirin/day starting on Day 5 following the intervention if platelets were >100 × 10^3^/µL. Despite aspirin treatment, one patient developed TIPS thrombosis. Platelet function results of this patient were not conspicuous or different compared to the other patients on any days tested (Figure S1). The most likely reason was an unfavorable placement of the TIPS device, as depicted in Figure S1. Such an explanation has also been proposed by others [8,42].

Since the usage of covered stents instead of bare metal stents, TIPS thrombosis has become a rather infrequent complication [43,44]. Nevertheless, antithrombotic treatment, either by anticoagulation or platelet inhibition, after TIPS implantation is still a widely used practice but varies tremendously among German hospitals [9]. Currently, there is no consensus on the requirement, choice and duration of antithrombotic agents for the management of cirrhotic patients following TIPS [45].

The results of our study suggest that pharmacological inhibition of platelet function may not be necessary to maintain stent patency in patients with decompensated cirrhosis and TIPS. However, this contention requires confirmation in larger studies.

Finally, we have to consider several limitations of our mono-center study. Firstly, due to the inclusion/exclusion criteria, the trial has been restricted to patients with platelet counts > 100 × 10^3^/µL. Therefore the findings cannot readily be translated into cirrhotic patient populations with a higher degree of thrombocytopenia. Secondly, the number of patients is small indeed. Thus, our trial rather resembles a preliminary study and does not allow drawing firm conclusions. As the incidence of TIPS thrombosis has decreased upon the use of covered stents, a very high number of patients would be required to examine the efficacy (and safety) of aspirin treatment in a placebo-controlled randomized trial after TIPS implantation. Therefore, we have used an exploratory approach to assess platelet function prior to aspirin treatment and also to study the drug effect on platelets in a follow-up of this setting. In addition, we decided not to increase the number of study patients due to the uniform findings with respect to the impaired aggregation responses obtained here using two different techniques to assess platelet function ex vivo (LTA, WBIA). Thirdly, the platelet count was not determined in portal-vein blood samples, but it can be assumed that blood counts in the portal, liver and peripheral veins do not differ. Fourthly, patients received aspirin starting on Day 5 following TIPS implantation. Thus, we cannot differentiate whether the decreased P-selectin surface expression upon stimulation in vitro obtained on Day 30 was influenced by aspirin, the beneficial effect of TIPS implantation on reducing portal hypertension, or a combination of both. Fifthly, healthy subjects were not age-matched with patients. We cannot exclude that the difference in age may have affected platelet function results. Eventually, a randomized, placebo-controlled trial of cirrhotic patients undergoing TIPS is required to test the rationale of pharmacological platelet inhibition, using patency or stent thrombosis as an end-point.

## 5. Conclusions

The present study demonstrates impaired platelet aggregation in patients with decompensated cirrhosis prior to and after TIPS implantation. Along with our findings, it appears to be highly questionable whether inhibiting platelet function by aspirin following TIPS intervention presents an appropriate strategy to prevent TIPS thrombosis. Indeed, the results of our study challenge the usefulness of aspirin as an antiplatelet regimen for cirrhotic patients following TIPS implantation.

## Figures and Tables

**Table 1:** Clinical and laboratory parameters of the study population.

Parameter	Patients D0 (n = 13)	Healthy Subjects (n = 9)
Male	10 (76.9%)	7 (77.8%)
Age (y)	61 ± 13	40 ± 5
Etiology		
Alcoholic	11 (84.6%)	
Hemochromatosis	1 (7.7%)	
Primary biliary cirrhosis	1 (7.7%)	
Indication for TIPS implantation		
Recurrent ascites	8 (61.5%)	
Secondary prophylaxis of variceal bleeding	5 (38.5%)	
**Clinical scores**		
Child–Pugh Score	7 (6–9)	
A	5 (38.5%)	
B	7 (53.8%)	
C	1 (7.7%)	
FIPS ^1^	0.07 (−0.78–0.53)	
Low risk	12 (92.3%)	
High risk	1 (7.7%)	
**Laboratory parameters**		
Hemoglobin (g/dL)	10.8 ± 1.8	
White blood cell count (×10^3^/µL)	7.9 ± 2.6	
Platelets (×10^3^/µL)	173 ± 50	
INR ^2^	1.26 ± 0.12	
PTT ^3^	38 ± 6	
Creatinine (mg/dL)	0.9 (0.7–1.5)	
Bilirubin (mg/dL)	1.4 (1.0–2.6)	
Albumin (g/L)	30 (28–35)	
AST ^4^ (U/L)	62 ± 24	
ALT ^5^ (U/L)	27 (22–54)	

^1^ FIPS, Freiburg index of post-TIPS survival; ^2^ INR, international normalized ratio; ^3^ PTT, partial thromboplastin time; ^4^ AST, aspartate aminotransferase; ^5^ ALT, alanine aminotransferase.

## Data Availability

The data are available upon reasonable request.

## Supplementary Materials

The following supporting information can be downloaded at: https://www.mdpi.com/article/10.3390/biomedicines11072057/s1, Figure S1: Analyses of platelet aggregation in a patient who developed TIPS thrombosis; Figure S2: Flow cytometric analysis of platelet activation markers and platelet-neutrophil complexes in patients with liver cirrhosis prior to TIPS implantation, using peripheral blood in comparison to portal-vein blood; Figure S3: Longitudinal analysis of platelet activation markers and platelet-neutrophil complexes in patients with liver cirrhosis and TIPS implantation.
