# Supplementary material for: Decreased Platelet Aggregation in Patients with Decompensated Liver Cirrhosis and TIPS Implantation
Source: Biomedicines. 2023 Jul 21;11(7):2057. doi: 10.3390/biomedicines11072057 (PMC10508239; doi:10.3390/biomedicines11072057)
Supplement: Supplementary file 1 [file biomedicines-11-02057-s001.zip › biomedicines-2491385-supplementary.pdf]

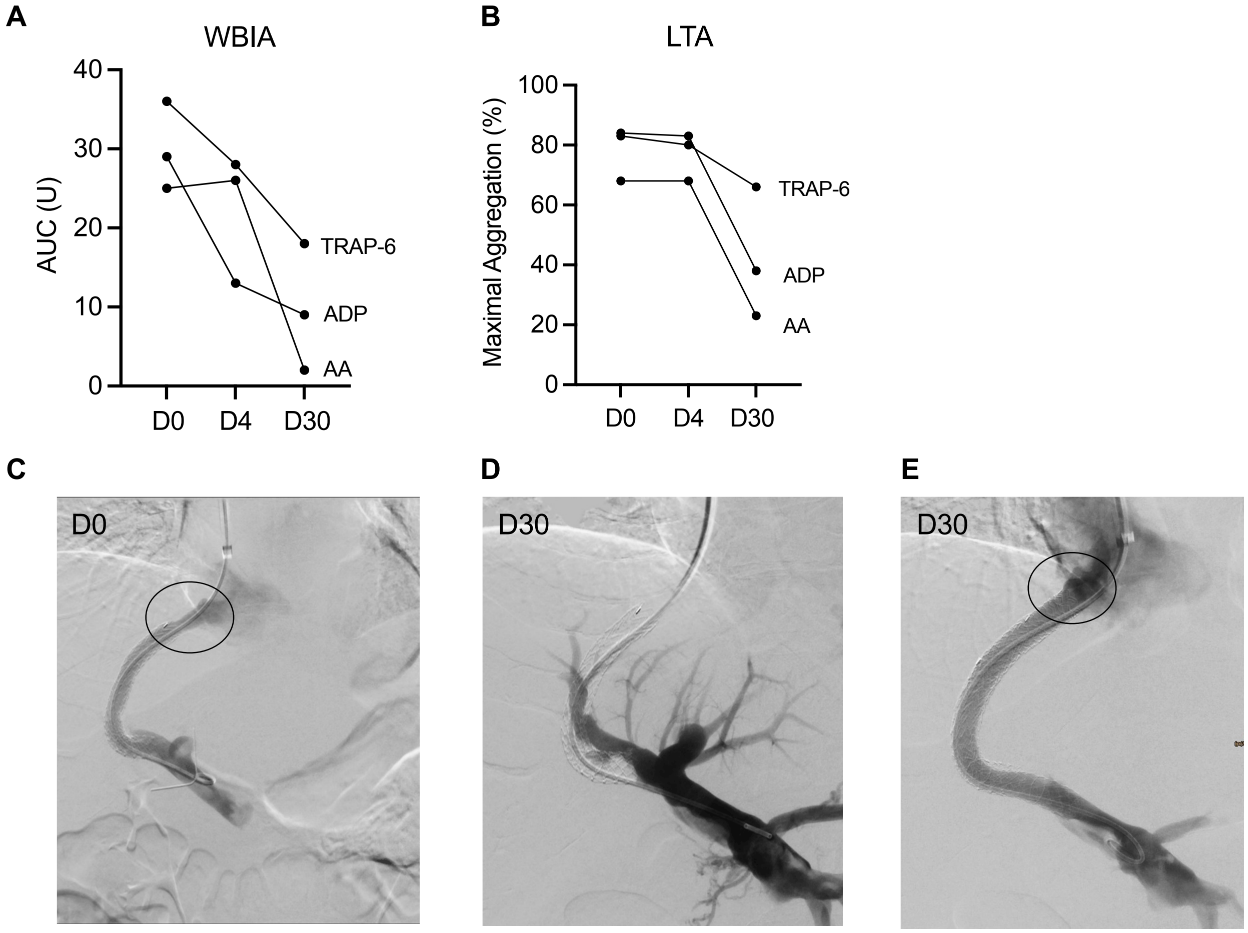

Figure S1: Analyses of platelet aggregation in a patient who developed TIPS thrombosis. Platelet aggregation was assessed in hirudinized blood (1:2 diluted) (**A**) and citrated blood-derived platelet-rich plasma (**B**) in a patient who developed TIPS thrombosis. Angiographic assessment of the inserted TIPS showed sufficient blood flow through the TIPS stent, although it has been placed too distant to the right atrium (circle, **C**). As color doppler ultrasound suspected TIPS dysfunction on D30, angiographic control was performed, and TIPS thrombosis was confirmed (**D**). After a stent-in-stent placement with a proximal position close to the right atrium (circle), blood flow through the stent was restored (**E**). AA, arachidonic acid; ADP, adenosine diphosphate; AUC, area under the curve; D0, Day 0; D4, Day 4; D30, Day 30; LTA, light transmission aggregometry; TRAP-6, thrombin receptor activating peptide 6; WBIA, whole-blood impedance aggregometry

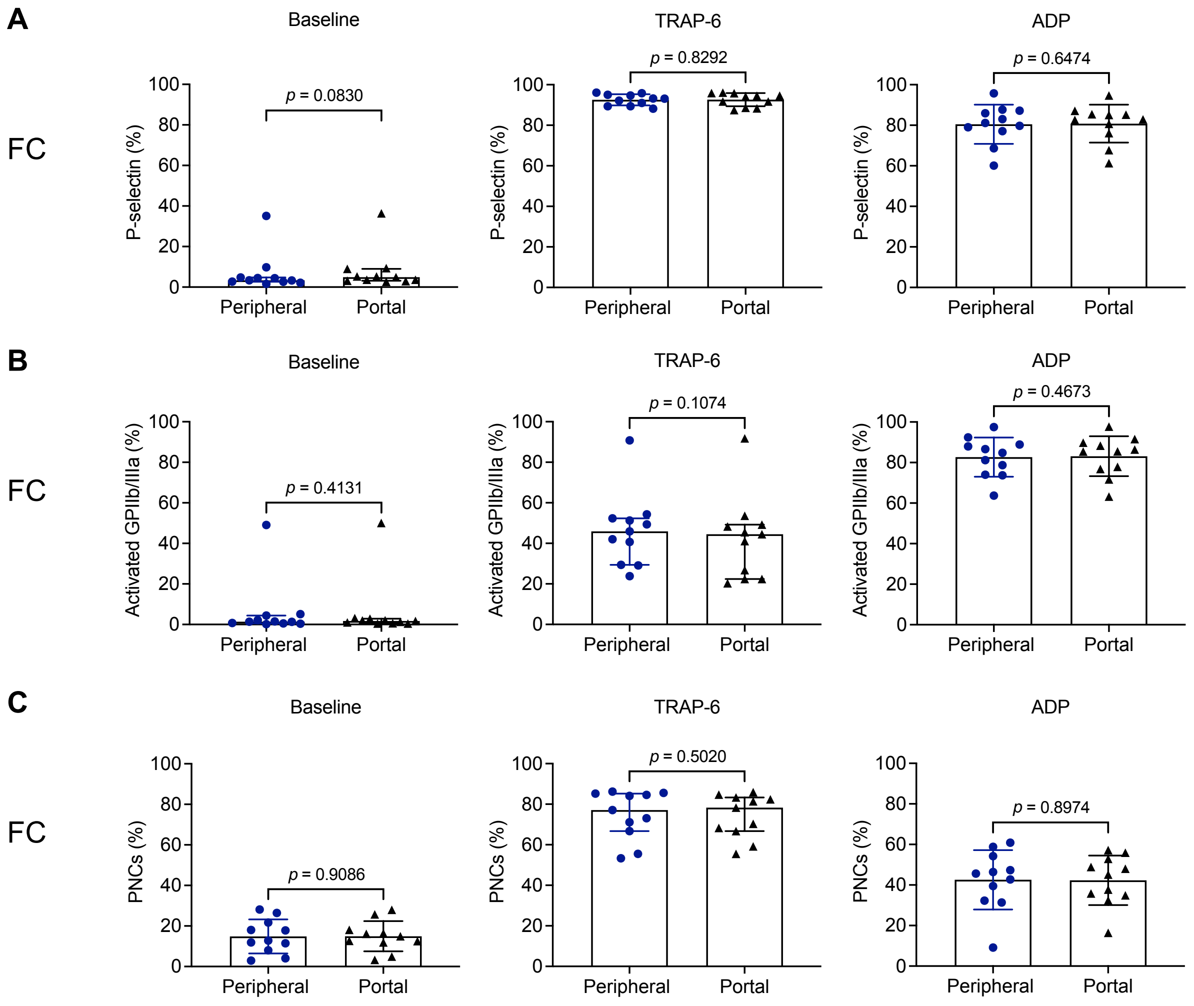

Figure S2: Flow cytometric analysis of platelet activation markers and platelet-neutrophil complexes in patients with liver cirrhosis prior to TIPS implantation, using peripheral blood in comparison to portal-vein blood. Surface expression of P-selectin (A), activated GPIIb/IIIa (B) and PNCs (C) was determined in citrated blood (1:6 diluted) from patients with liver cirrhosis (n = 11, blue circles: peripheral blood, black triangles: portal-vein blood) at baseline or in response to TRAP-6 or ADP using flow cytometry. Results are presented as the percentage of positive cells. ADP, adenosine diphosphate; FC, flow cytometry; PNCs, platelet–neutrophil complexes; TRAP-6, thrombin receptor activating peptide 6

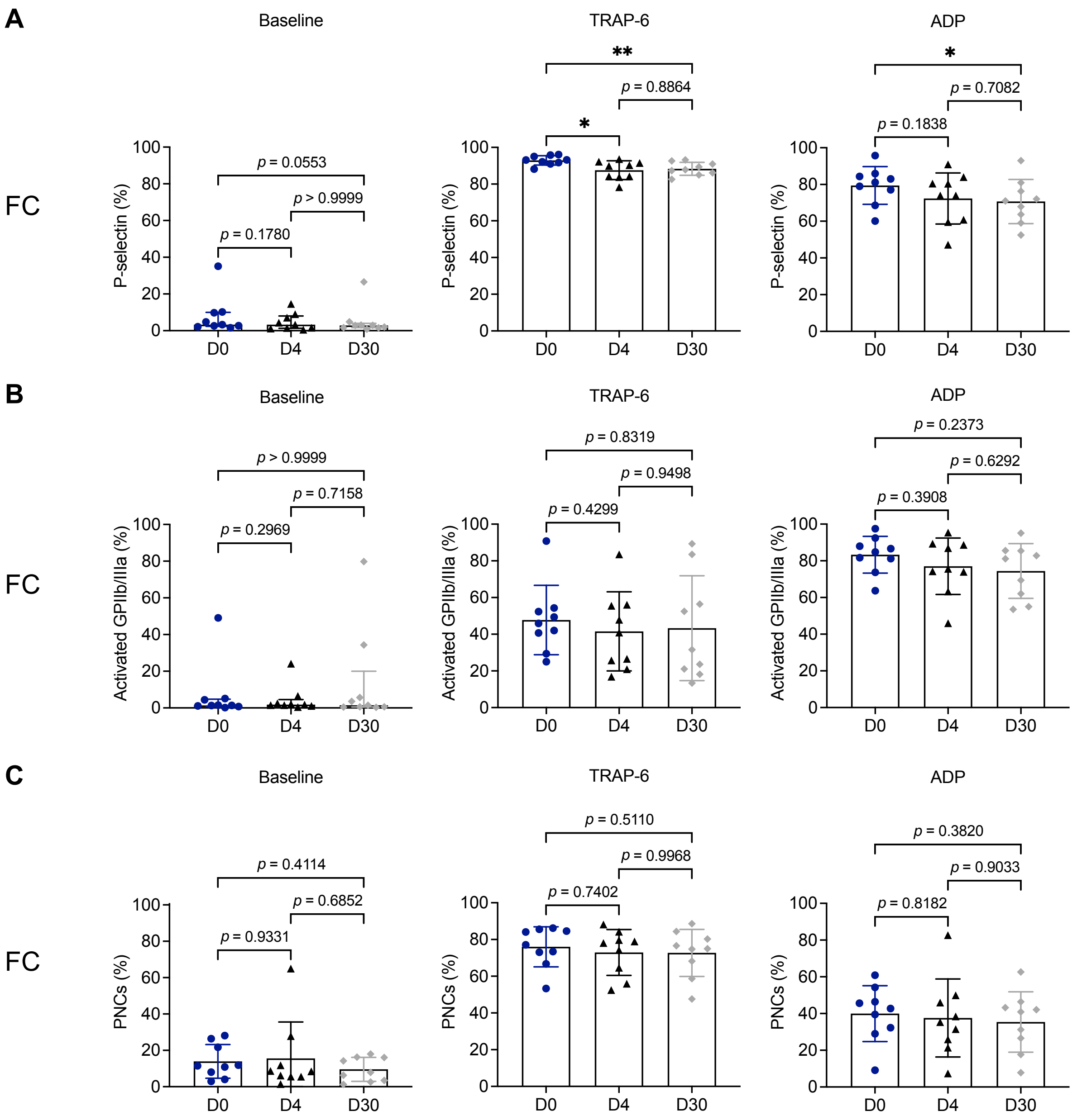

Figure S3: Longitudinal analysis of platelet activation markers and platelet-neutrophil complexes in patients with liver cirrhosis and TIPS implantation. Surface expression of P-selectin (A), activated GPIIb/IIIa (B) and PNCs (C) was determined in citrated blood (1:6 diluted) from patients with liver cirrhosis (n = 9) prior to (blue circles: Day 0) and after TIPS implantation (black triangles: Day 4, grey diamonds: Day 30) at baseline or in response to TRAP-6 or ADP using flow cytometry. Results are presented as the percentage of positive cells. * $p < 0.05$; ** $p < 0.01$. ADP, adenosine diphosphate; FC, flow cytometry; D0, Day 0; D4, Day 4; D30, Day 30; PNCs, platelet–neutrophil complexes; TRAP-6, thrombin receptor activating peptide 6
